# Protein homologous cores and loops: important clues to evolutionary relationships between structurally similar proteins

**DOI:** 10.1186/1472-6807-7-23

**Published:** 2007-04-10

**Authors:** Thomas Madej, Anna R Panchenko, Jie Chen, Stephen H Bryant

**Affiliations:** 1 Computational Biology Branch, National Center for Biotechnology Information, Building 38A, National Institutes of Health, Bethesda, Maryland 20894, USA

## Abstract

**Background:**

To discover remote evolutionary relationships and functional similarities between proteins, biologists rely on comparative sequence analysis, and when structures are available, on structural alignments and various measures of structural similarity. The measures/scores that have most commonly been used for this purpose include: alignment length, percent sequence identity, superposition RMSD and their different combinations. More recently, we have introduced the "Homologous core structure overlap score" (HCS) and the "Loop Hausdorff Measure" (LHM). Along with these we also consider the "gapped structural alignment score" (GSAS), which was introduced earlier by other researchers.

**Results:**

We analyze the performance of these and other conventional measures at the task of ranking structure neighbors by homology, and we show that the HCS, LHM, and GSAS scores display considerably improved performance over the conventional measures of sequence or structural similarity.

**Conclusion:**

The HCS, LHM, and GSAS scores are easily computable quantities that allow users of structure-neighbor databases to more easily identify interesting structural similarities between proteins.

## Background

Discovering structural similarity between proteins or their parts can shed light on their evolutionary relationships. Since evolutionarily related proteins are highly likely to share common aspects of function, measures of structural similarity that can distinguish between related and unrelated proteins can be particularly useful for protein functional annotation. Discerning homology between structurally similar proteins is complicated by the fact that proteins can have very similar structures but be quite diverse in sequence and there is a belief that structurally similar proteins can arise due to either convergent or divergent evolution. Sequence similarity searches very often fail to rank correctly structurally similar but remotely related proteins, and also have limited ability to distinguish structurally similar evolutionarily related proteins from the unrelated ones.

Structure-structure alignment algorithms are the best known methods to produce evolutionarily correct alignments between remotely related proteins [1]. All structure alignment methods require a target scoring function to optimize and a large variety of different scoring functions have been developed in this connection. Unlike sequence alignment methods which all use conventional amino acid substitution matrices for scoring, there is no consensus among different statistical scores used in structure alignment algorithms. Moreover, these algorithms can be successful at finding a reasonable alignment but can fail at ranking good alignments ahead of the problematic ones using the target scoring functions [2,3].

To compensate for this effect researchers have developed a number of different structural similarity measures to rank structurally similar proteins [3-6] or to produce meaningful clustering based on structural comparison [5,7,8]. Some commonly used measures are: length of the alignment, superposition RMSD, percent sequence identity and their various algebraic combinations. It has been shown that these particular measures of structural similarity have a limited success at ranking of structurally similar proteins and distinguishing structurally similar related proteins from unrelated ones [9-14]. As an improvement Matsuo and Bryant introduced the "Homologous Core Structure overlap score" (HCS score) [9] which is calculated as a fraction of a "homologous core" that is covered by a structural alignment, where the homologous core is determined by that part which is conserved in structural superposition of homologous proteins. More recently, Panchenko and Madej have introduced the "Loop Hausdorff Measure" (LHM), which measures the (average) amount of deviation between the loop regions in a pair of superposed protein structures [15]. This new similarity measure is analogous to the traditional RMSD, but has the advantage that it can be applied to the corresponding but non-alignable regions in the two structures. It was shown that the loop regions in homologous proteins display a certain level of structural conservation, and the LHM is very sensitive in detecting subtle differences between protein structures otherwise unrecognized by conventional similarity scores [15].

It should be noted that there are different ways to assess the ability of structural similarity scores to rank structurally similar homologs with respect to their relatedness. For example, Yang and Honig showed that there exists a correlation between the significant sequence similarity and their measure of structural distance even in the twilight zone of sequence similarity, so that high values of structural distance would correspond to the high probability that proteins are related by common descent and vice versa [15]. Similarly, in a recent paper sequence-structure relationships for homologous proteins have been analyzed for different families/folds and it has been found as a corollary to other results that the quality of linear sequence-structure correlation varies depending on the structure similarity scores used in the study [16].

In this paper we apply a ROC analysis to the problem of ranking of structure neighbors with respect to homology, where we decide evolutionary relationships based on the SCOP database [17]. We use the "superfamily" level in the SCOP database hierarchy because almost certainly the proteins in a SCOP superfamily display probable homology and common functionality. Using SCOP as a standard for evolutionary relationships in this study is problematic since other researchers have produced convincing arguments for homology between proteins in different SCOP superfamilies. However, the particular cases where difficulties would be expected are largely confined to families involving "superfolds", such as TIM barrels or Rossmann folds, and the analyses to convincingly establish homology are rather complicated. Thus, although evolutionarily related proteins may be excluded because SCOP concluded there was insufficient evidence for homology, it remains the case that the superfamily members of a query protein are those for which there is clear and convincing evidence of functional relatedness. Based on structure-structure superpositions we define different structure similarity scores and test their performance on the difficult benchmark of VAST neighbors (structurally similar protein domains found by the VAST algorithm [18]). We find that from among the scores we consider, the LHM, HCS, and GSAS score ("gapped structural alignment score" introduced in [3]) exhibit the best performance, especially for the remotely related proteins.

## Results

For convenience we quickly summarize out methods. Further details are provided in the Methods section. A selection of conserved domain (CD) families were taken from the Conserved Domain Database (CDD). For each CD family a query (representative) structure was chosen and a list of similar structures (neighbors) was generated using the VAST algorithm. The lists were filtered by sequence identity to reduce redundancy. For a given query structure, those neighbors on its list were considered to be "true postives" if and only if they belong to the same superfamily as the query in the SCOP database. A given structural similarity measure/score can be used to rank the pairs of queries and neighbors, and for a chosen cutoff, we can compute the fractions of true positives (sensitivity) and false positives found at or above the cutoff. The fractions of true and false positives provide a basis for comparing the performance of the different similarity measures.

**Figure 1:**
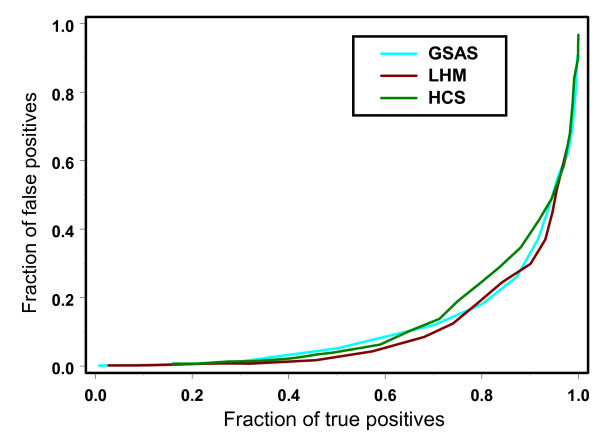
**Sensitivity curves for the three best-performing measures**. The fraction of correctly ranked homologous VAST neighbors (true positives, sensitivity) is plotted against the fraction of incorrectly ranked homologous VAST neighbors for similarity measures yielding the best performance: HCS (green)), LHM (red) and GSAS (cyan). True and false positive (error) rate values at each cutoff of similarity measures were averaged over protein families from the overall test set (152 families).

Table 1 shows the sensitivities of all eight similarity scores at two given error (false positive) rates (1% and 5%). As can be seen from this table the LHM, GSAS and HCS measures demonstrate greater sensitivity than the conventional measures of structural and sequence similarity. For example, at the 1% error rate, LHM detects more than twice as many true positives on average as RMSD and fraction aligned, and more than 1.5 times as many true positives as percent identity. In Figure 1 we plot the sensitivity curves for the three scores which perform the best (GSAS, LHM and HCS). It is apparent from this figure that the LHM curve lies lower than the curves corresponding to HCS and GSAS indicating that LHM outperforms on average these two other measures for the overall test set of 152 families.

**Figure 2:**
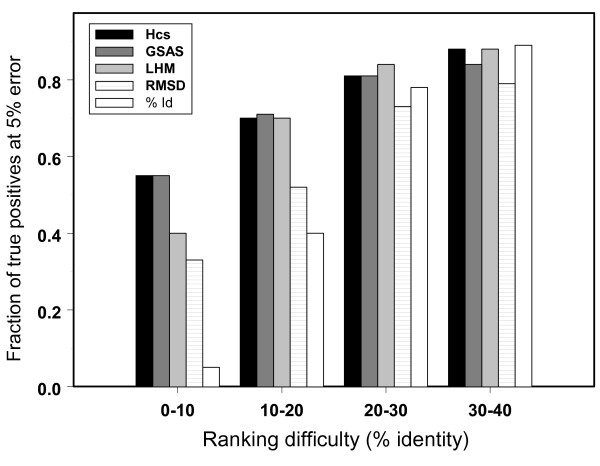
**Performance on families of differing degrees of difficulty**. The barplot shows the sensitivity at 5% error rate for each bin of ranking difficulty. Ranking difficulty is estimated as an average percent identity between the query structure and non-redundant set of true positive structures (homologous VAST neighbors) for each CDD family. Each bin of percent identity contains at least five CDD families within a given range of ranking difficulty and sensitivity is averaged over the sensitivities of CDD families within a given bin. CDD families were chosen here as those with at least 20 non-redundant VAST neighbors. There are 13 CDD families in the 0–10% bin; 52 in the 10–20% bin; 21 in the 20–30% bin; and 11 in the 30–100% bin (97 CDD families altogether).

It is also of interest to compare the performance of the different measures with respect to the ranking difficulty. To estimate the ranking difficulty for each CDD family we take the average percent identity between its query structure and the non-redundant set of true positive structures (homologous structure neighbors). There is a broad distribution of sensitivity values across the different degrees of ranking difficulty as shown in Figure 2, implying that some domain families are easier to recognize than others. Queries which have closely related structure neighbors show higher sensitivity and vice versa, this trend is apparent for all similarity scores used in the study. It should be noted that this analysis was done on a smaller test set of 97 families which had enough family members (at least 20) to make the calculation of sensitivities per family more reliable. We also note that 13 of the CDD families are in the most difficult bin (no more than 10% average sequence identity) and 52 are in the second most difficult bin, where the average sequence identity ranges from 10–20%. Thus, 65 of the 97 CDD families may be considered to be well within the zone of sequence similarity where homology is hard to ascertain.

Comparing the different scores, it is clear from this figure that HCS, GSAS and LHM exhibit better sensitivity in the twilight zone of sequence similarity below 30% compared to other scores used in this study. Moreover, HCS and GSAS outperform the others in the most difficult cases below 10% of sequence identity. This is not surprising, for example, GSAS represents a combination measure using alignment length, RMSD and the number of unaligned gapped regions. It is not unexpected that a combination measure should do well. As was shown earlier, a linear combination of alignment-based structural score (RMSD) and loop-based structural score (LHM) had a much better performance compared to each of the scores used separately [19].

The HCS scores use CD core models which have been determined by careful manual alignment curation using both sequence and structure data. From Figure 2 it is quite clear that recognizing this common conserved core is a powerful method for inferring homology and functional similarity in the most difficult cases. For example, the Class I amino acyl-tRNA synthetase (aaRS) catalytic core domain (cd00802) using the HCS score yields a sensitivity of 0.79 at the 5% error rate whereas the sensitivities obtained with other measures are substantially lower (0.44, 0.26, 0.67, 0.23, and 0.44 with percent identity, RMSD, LHM, fraction aligned, and GSAS respectively). The aaRS catalytic core domain has 56 non-redundant structure neighbors of which 12 are in the same SCOP superfamily, with an average of about 10% sequence identity. The aaRS structural core is based on the Rossmann fold and is well-conserved with a number of functionally important sites located at different core regions. These include a pair of ATP-binding sites with important sequence/structural motifs (the "HIGH" and "KMSKS" motifs) that are characteristic for class I aaRS and included in the core model. Such features cause the HCS score to rank the SCOP superfamily members in this family more highly than the other numerous Rossmann folds with more remote evolutionary relationships and less functional similarity.

The preceding analysis concerns the average performance of the various measures. However, in practice most researchers will be interested in particular protein families, and so we should also investigate what happens in specific cases. To do so, we first further limit the test set to those CDD families with at least 10 true positives and 10 false positives among their non-redundant structure neighbors; there are 44 such CDD families altogether. We found that there are 20 CDD families for which at least one similarity score (LHM, HCS or GSAS) had a sensitivity higher than 80% at the 5% false positive rate. On the other hand, there are seven CDD families for which all three scores have a sensitivity of less than 50% at the 5% false positive rate (Table 2).

It is apparent from Table 2 that the seven "difficult" CDD families involve folds that span a broad range of sequence, function, and phylogenetic diversity and are often referred to as "superfolds". It is certainly to be expected that the measures we consider should encounter difficulty in the correct evolutionary ranking for structure neighbors of such families. Most of these superfolds have protein cores which are very well conserved among all diverse members of these folds due to stability, foldability, or other requirements. Certainly, subtle structural/sequence features or motifs that may provide clues to evolutionary relationships are not all included in our CDD-derived core models. Moreover, as was shown previously there is evidence that all proteins from certain superfolds have a common ancestor and are all therefore possibly homologous (by definition) [19-21].

We also compared the measures over the four different major SCOP fold classes, at the 1% and 5% error rates. These results are available as supplementary data [see Additional file 1] and via the internet at [22].

## Discussion and conclusions

Most users of structure comparison methods will be interested mainly in those similarities which may shed light on the function of their query protein, and hence are primarily interested in the homologous neighbors. The scoring functions of the various structure comparison algorithms are useful for ranking the neighbors, however, the rankings they produce are much less than perfect, particularly in the "twilight zone" of similarity. This is not surprising. For example, VAST scores and E-values are devised to recognize fold similarity for simplified vector models of protein structures. Such vector models capture only gross similarities between the spatial arrangements of secondary structure elements in proteins, and one would suspect that such scores are too coarse-grained to do well at ranking homologs. In fact, in a recent paper by Sierk and Pearson [2], the authors have found that the scoring schemes for a number of different structure alignment algorithms do not perform appreciably better at detecting homologs than normalized RMSD.

We have compared the different similarity measures via an ROC analysis using the SCOP superfamily level as our definition of functional similarity/evolutionary relatedness. It is arguable as to whether or not this definition is inclusive enough, for example, the current consensus seems to be that almost all the TIM barrels are homologous, although SCOP groups them into 31 distinct superfamilies (SCOP release 1.69). Nonetheless, if we are to rank by functional relatedness, the SCOP superfamily members of a given query protein are surely more closely related than other similar structures.

In this paper we have presented scores such as the "Loop Hausdorff Measure" and "Homologous Core Structure", which are superior to the conventional structural similarity measures (percent identity, normalized RMSD, and fraction aligned) at the task of ranking homologous, structurally similar proteins. A combination measure such as the "Gapped Structural Alignment Score" also performs well. We have shown (see Figure 2) that in the more difficult cases, where the sequence identity between the query and its neighbors is low (30% or less), the LHM, HCS, and GSAS scores clearly produce the better rankings.

Protein structure comparison continues to be an active area of research and highly interesting new methods and studies continue to appear, e.g. [23-25]. However, we have focussed on the LHM and HCS scores in this paper since they are of intrinsic biological interest. Indeed, the LHM and HCS are easily interpretable as they quantify divergence of loop regions and conservation of structure, respectively, so that consideration of these measures can lead to deeper insights into structural evolutionary relationships.

More detailed case-by-case examination of individual CDD families shows that none of the scoring schemes works perfectly. For this reason it is important to make available several of the best-performing ranking schemes, and we are currently working on adding the LHM and HCS scores to the VAST web server and VAST Search web service. Improved rankings may reduce the number of neighbors in the "twilight zone", where it is difficult to discern homology/functionality, however, users will still need to examine other evidence such as sequence/structure conservation at functional sites in order to reach a firm conclusion.

## Methods

### Description of the test set

In this paper we design a test set of structure-structure neighbors with recorded "homologous" relationships between them, which are defined as those structurally similar proteins belonging to the same SCOP (version 1.67) superfamily category [17]. A homologous core model for HCS calculation was taken from the curated Conserved Domain Database (CDD) alignments [26]. Curated CDD alignments have been refined using three-dimensional structures and structure-structure alignments and core regions in CDD alignments are defined as those conserved/aligned among all family members of a given conserved domain (CD). We start our analysis with a set of 362 curated alignments from CDD version 2.00 [27], the current version of which is available at [28]. The chosen CDD alignments correspond to the top node ("parent") alignments in the hierarchy of CDD families. This means that they represent more general families, whereas nodes that occur below them in the hierarchy represent more specific families.

It is necessary to filter our initial list of CDs in order to ensure mutual consistency between the CD core models, MMDB domains [29](which are automatically generated and used by the structure comparison method), and SCOP domains [17]. In order to do this we first exclude small CD core models from further consideration (those with less than 50 residues) since these core models are often too general to be able to find specific family members. For each remaining CDD alignment we choose one representative structure so that the CDD footprint on this structure and corresponding MMDB domain/chain boundaries are consistent to a degree of 80% mutual overlap and, simultaneously, the mutual overlap between MMDB domain/chain boundaries and SCOP domain boundaries of this structure is at least 80%. In this case we can say that the CD core model is consistent with the corresponding representative MMDB domain. The "footprint" here is defined as a region on a representative structure between the first and the last residues aligned in the CD. All MMDB domains and a full length chain of the representative structure (disregarding chain discontinuous domains) are checked and the domain/chain with the maximum overlap is used as a representative for a given CD. The collection of all representative structures forms the set of queries.

All structure neighbors and structural alignments were obtained from the PubVast database, which contains the results of pairwise comparisons between all structures in the PDB, using the VAST algorithm [18]. At the next stage all structure neighbors for each query domain/chain were retrieved from the PubVast database and only those with more than 80% mutual overlap between VAST alignment and SCOP domain footprints were selected for further analysis. By doing this we can compare the SCOP classification for the query and its structure neighbors. In order to eliminate the redundancy among structure neighbors we use redundancy groups which were constructed previously by single-linkage clustering of MMDB chains based on BLAST E-value of 10^-40 ^or less. Only those structure neighbors belonging to distinct redundancy groups were counted as being different and those structure neighbors from the same redundancy group as the query were excluded from consideration.

True positives in our test set are defined as those neighbors having the same SCOP superfamily category as the query. At the end of this filtering procedure, 152 queries with corresponding CDD alignments were collected, each of them having at least one true positive entry satisfying all the above criteria. The list of queries together with the corresponding CDD families for HCS definition is available at [30].

### Evaluation of the measures – sensitivity analysis

We evaluate the different measures of structural similarity based on correct detection and ranking of homologous structure neighbors (true positives) versus non-homologous structure neighbors (false positives). For a given query and its structure neighbor list, we descend the list and calculate the true positive and false positive ratios at each similarity measure cutoff. This produces a sensitivity curve. The true positive ratio (sensitivity) is defined as the number of detected true positives divided by the overall number of true positives of the given CDD family. The false positive ratio is simply the number of found false positives divided by the overall number of false positives of the given CDD family. To compare the sensitivities for different scoring schemes we use the sensitivity values found at 1% and 5% of false positive rate (error rate). The higher the sensitivity at a given error rate, the better the performance of a given similarity measure. Since different CDD families have different numbers of true and false positives there can be a certain bias towards large families. To compensate for this bias we plot the sensitivity curves averaged over all CDD families (Figure 1). We also calculate the sensitivity at the 5% error rate separately for each CDD family (Figure 2), in which case a test set of 97 CDD families has been used with at least 20 non-redundant structure neighbors.

### Measures of sequence and structural similarity

The HCS overlap score for each pair of aligned structures (i.e. alignment of a query to each structure neighbor) was calculated as a ratio between the number of residues from the CD core model that were also included in the structure-structure alignment and the total number of residues in the conserved core model (both of these instances of "core model" refer to the query structure). This quantity was originally defined in [9]. The loop structural similarity measure (Loop Hausdorff Measure) was calculated as described previously [19]. Informally, LHM is the average amount by which the corresponding loop regions (regions between aligned secondary structure elements) differ from each other in a pair of superimposed structures. The structural neighbors having more than 25% of the loop residues with missing coordinates for Cα atoms were not considered in the analysis for LHM. Root mean squared deviation (RMSD) was computed for the structure alignments using the superposition algorithm due to McLachlan [31]. "Fraction aligned" was calculated as the ratio between the number of residues aligned and the total number of residues in the smaller of the two domains.

The structural similarity measures RMSD and LHM were normalized by dividing by the square root of the number of aligned residues in order to eliminate dependence on the number of residues and protein size. Non-normalized conventional measures of structural similarity have yielded weaker performance in the current sensitivity analysis (not shown) and other analyses reported earlier [16]. To compare the abovementioned similarity scores with the measures used by other authors we also calculated structural similarity measures used by Kolodny et al in their evaluation of different structure-structure alignment methods [3]. Two of these measures (SI and MI) represent an algebraic combination of RMSD and "fraction aligned", while the third one (GSAS) depends not only on RMSD and the alignment length but also on the number of gaps in the structure-structure alignment.

We do not expect that the results described are dependent in any essential way on the particular algorithms or databases used in this study. For example, the LHM is not sensitive to the particular secondary structure assignment algorithm that is used, because it is an average over maximum deviations in the loop regions between two superposed stuctures. The precise secondary structure element definitions do not usually affect this. The major factor that could cause our results to be unreliable is if the structure-structure superpositions that we used were grossly inaccurate. However, the statistics on the superposition RMSDs for the alignments in this study indicate that the alignments must be very reasonably accurate. For our collection of alignments, the average superposition RMSD was 2.7 Å with a standard deviation of under 0.9 Å. The largest RMSD was 6.2 Å and only 3% of the alignments were under 1.0 Å while less than 1% were over 5.0 Å in RMSD.

### Availability of data and programs

Pre-computed VAST structure neighbors are accessible by PDB codes and chain/domain identifiers via the internet at [32]. The structure neighbor list may be sorted by various options, including alignment length, percent sequence identity, and RMSD. These sorting options are also available for viewing neighbor lists in the VAST Search web server. The VAST Search web server computes the structure neighbors for user-submitted files in the PDB format; it is located at [33]. The LHM and GSAS scores have been added to the pre-computed VAST neighbors. The work to add the HCS score for the pre-computed neighbors, and all three scores to the VAST Search web server, is in progress. All computer programs used in this work are freely available upon request.

## Authors' contributions

TM and ARP conceived and carried out the study and drafted the manuscript. JC did necessary programming for the VAST structure neighbors database. SHB contributed to the interpretation of the data. All authors read and approved the final manuscript.

## Supplementary Material

Additional file 1 Supplementary tables. Sensitivity values for similarity measures studied, with domain families grouped by fold classes. Click here for file

## Figures and Tables

**Table 1:** Sensitivity values estimated from curves. Sensitivity values estimated from the curves (Figure 1) at 1% and 5% error rates (fraction of false positives) are listed for different similarity measures: loop Hausdorff measure (LHM), HCS score (HCS), gapped structural alignment score (GSAS), percent aligned (%aln), percent identity (%id), root mean square deviation (RMSD), and two other structural similarity measures (SI and MI) from [3].

	**LHM**	**HCS**	**GSAS**	**% aln**	**% id**	**RMSD**	**MI**	**SI**
**1% error rate**	0.36	0.24	0.26	0.14	0.23	0.17	0.15	0.07
**5% error rate**	0.59	0.54	0.49	0.45	0.44	0.44	0.43	0.31

**Table 2:** Difficult families. Difficult families for all of the measures. For these seven CDD families all of the six measures had sensitivities of under 0.50 at the 5% error rate.

**CDD acc**	**CDD short name**	**SCOP fold class**
cd00945	Aldolase_Class_I	TIM β/α barrel
cd00529	RuvC_resolvase	Ribonuclease H-like motif
cd01120	RecA-like_NTPases	P-loop containing nucleoside triphosphate hydrolases
cd00079	HELICc	P-loop containing nucleoside triphosphate hydrolases
cd00453	FTBP_aldolase_II	TIM β/α barrel
cd00102	IPT	Immunoglobulin-like β-sandwich
cd00234	RPA14	OB-fold
