# Supplementary material for: Protein homologous cores and loops: important clues to evolutionary relationships between structurally similar proteins
Source: BMC Struct Biol. 2007 Apr 10;7:23. doi: 10.1186/1472-6807-7-23 (PMC1852803; doi:10.1186/1472-6807-7-23)
Supplement: Additional file 1 — Supplementary tables. Sensitivity values for similarity measures studied, with domain families grouped by fold classes. [file 1472-6807-7-23-S1.doc]

**Supplementary tables** for “Protein homologous cores and loops: important clues to evolutionary relationships between structurally similar proteins”, by T. Madej, A.R. Panchenko, et al.

The tables present the sensitivity values for the similarity measures studied in the paper, with the CD families grouped by the four main SCOP fold classes, and for the 1% and 5% error rates.

Numbers of CDs for the SCOP classes

**All β**: 34

**α/β**: 51

**α+β**: 32

**All α**: 25

| 0.01 err | **LHM** | **HCS** | **GSAS** | **%aln** | **%id** | **RMSD** | **MI** | **SI** |
| --- | --- | --- | --- | --- | --- | --- | --- | --- |
| **All β** | 0.26 | 0.30 | 0.06 | 0.02 | 0.17 | 0.07 | 0.02 | 0.01 |
| **α/β** | 0.35 | 0.24 | 0.23 | 0.22 | 0.14 | 0.22 | 0.34 | 0.28 |
| **α+β** | 0.60 | 0.67 | 0.66 | 0.44 | 0.41 | 0.27 | 0.34 | 0.11 |
| **All α** | 0.27 | 0.04 | 0.22 | 0.11 | 0.26 | 0.13 | 0.30 | 0.13 |

| 0.05 err | **LHM** | **HCS** | **GSAS** | **%aln** | **%id** | **RMSD** | **MI** | **SI** |
| --- | --- | --- | --- | --- | --- | --- | --- | --- |
| **All β** | 0.50 | 0.44 | 0.36 | 0.20 | 0.49 | 0.29 | 0.21 | 0.20 |
| **α/β** | 0.53 | 0.50 | 0.51 | 0.45 | 0.31 | 0.47 | 0.48 | 0.43 |
| **α+β** | 0.77 | 0.81 | 0.81 | 0.74 | 0.57 | 0.54 | 0.57 | 0.39 |
| **All α** | 0.61 | 0.32 | 0.44 | 0.41 | 0.45 | 0.39 | 0.55 | 0.36 |
